# Compact all-fiber source of coherent linearly polarized octave-spanning supercontinuum based on normal dispersion silica fiber

**DOI:** 10.1038/s41598-019-48726-9

**Published:** 2019-08-23

**Authors:** Karol Tarnowski, Tadeusz Martynkien, Paweł Mergo, Jarosław Sotor, Grzegorz Soboń

**Affiliations:** 1 Department of Optics and Photonics, Faculty of Fundamental Problems of Technology, Wrocław University of Science and Technology, Wybrzeże Wyspiańskiego 27, 50-370 Wrocław, Poland; 2 Laboratory of Optical Fiber Technology, Maria Curie-Sklodowska University, pl. M. Curie-Sklodowskiej 3, 20-031 Lublin, Poland; 3 Laser & Fiber Electronics Group, Faculty of Electronics, Wrocław University of Science and Technology, Wybrzeże Wyspiańskiego 27, 50-370 Wrocław, Poland

**Keywords:** Fibre lasers, Supercontinuum generation

## Abstract

We report the generation of coherent octave-spanning supercontinuum in an all-fiber system, without any free-space optical components. The setup uses the femtosecond fiber laser as a pump and an all-normal dispersion microstructured fiber as a medium for supercontinuum generation. The generated spectrum is characterized both experimentally and numerically and shows a broad bandwidth (1.1−2.2 μm), a high signal to noise ratio reaching 100 at maximum, a high coherence (closing to 1), linear polarization and average output power up to 57 mW. The source is characterized by exceptional simplicity and does not require any alignment (the nonlinear fiber is spliced to the pump) which finally opens the path to outside-lab applications of supercontinuum radiation.

## Introduction

The development of fiber-based supercontinuum (SC) sources took off in the year 2000, when Ranka *et al*. demonstrated a suitability of microstructured fibers (MOFs) for SC generation^1^. An important step was performed few years later by utilizing fibers with all-normal dispersion (ANDi) for SC generation. The works by T. Hori *et al*. and N. Nishizawa *et al*. showed that SC generated in an ANDi fiber is characterized by higher degree of coherence and lower relative intensity noise (RIN) than SC obtained in conventional, anomalous-dispersion fibers^2,3^. The ANDi-SC is also characterized by improved flatness, with maintained octave-spanning spectral width^3^. Generation of coherent SC under femtosecond pumping in ANDi fibers was also investigated by Heidt *et al*.^4^. They found that in this regime the SC is generated by a self-phase modulation (SPM) broadening^4^ and a subsequent optical wave breaking (OWB)^5^. In 2011, octave-spanning SC was generated in the ANDi microstructured silica fiber with a long wavelength limit reaching 1.5 μm^6^. Subsequently, non-silica fibers were used to shift ANDi SC towards mid-infrared^7,8^. Next, very broad mid-infrared SC spectra were generated in chalcogenide step index fibers^9,10^. In parallel, the effort was taken to reach the long wavelength edge of silica glass transmission window with ANDi SC. The germanium doped microstructured silica fibers allowed to shift the long wavelength limit of ANDi SC to 2.2 μm (with a pump at 1.55 μm)^11,12^ and later on beyond 2.5 μm (with a pump tuned in 1.8–2.4 μm range)^13^. Very recently, also a pure silica fiber with zero-dispersion wavelength at 1.8 μm enabling low-noise SC generation was reported^14^. The important advantage of silica-based fibers is a possibility to obtain a low-loss and robust splice to a fiber laser source. As a result, by connecting the fiber laser with the nonlinear fiber one can develop a compact all-fiber supercontinuum source, without using any bulk optics and optomechanics, and without the necessity of any alignment. This idea was already applied for a subpicosecond laser operating at 1.56 μm and a step-index silica fiber with zero dispersion wavelength at 1.59 μm^15^ which resulted in generation of a very broad supercontinuum covering the 1.12–2.24 μm spectral range. Likewise, an all-fiber system generating a 0.7–1.7 μm supercontinuum was developed by splicing a microstructured silica fiber with a 1.064 μm picosecond pump^16^. The obtained spectras’ coherences were limited, due to operation in both: normal and anomalous regimes of chromatic dispersion. A coherent supercontinuum source with the bandwidth of 624 nm was developed by Okamura *et al*.^17^. However, pump pulses generated in Er-based system were launched to the normal dispersion highly nonlinear fiber using bulk optics. Moreover, the single-mode fiber was used as an intermediate stage to convert laser pulse into Raman soliton centered at 1.67 μm, which was filtered with a low pass filter and used as the pump for SC generation process. The octave spanning supercontinuum was generated by Nozaki^18^ in a dispersion shifted fiber (the spectrum from 0.95 μm to 2.30 μm and in a normal dispersion fiber (the spectrum from 1.05 μm to 2.15 μm). However, the presented system is not all-fiber again.

Keeping the system entirely fiberized has multiple advantages. First of all, it is completely alignment-free. Secondly, it can be built entirely from polarization maintaining (PM) fibers, which ensures a linear polarization state at the output and invulnerability to external disturbances. Finally, it significantly reduces the costs of the system, by avoiding free-space optics (telescopes, objectives) and micrometer stages used for alignment. These are important features from the application point of view. To the best of our knowledge, coherent and all-normal dispersion generation of SC was not demonstrated in a fully fiberized compact system. Developing such compact and robust source could increase the scope of applications of ANDi-SC. The SC sources based on microstructured fibers were successfully applied for example in a confocal microscope^19^, in an optical coherence tomography^20^ and in the measurement of optical frequencies^21^.

In this work, we present an all-fiber source of coherent and linearly polarized octave-spanning supercontinuum generated in an all-normal dispersion fiber. We used a femtosecond Er-doped fiber laser as a pump source and spliced it directly with the SC fiber. Importantly, the silica SC fiber is fully compatible with telecom-grade single-mode fibers, so it does not require any sophisticated splicing techniques. The generated SC has an average power of 57 mW (at 45 MHz repetition rate) and spans over one octave (1.1–2.2 μm). We have confirmed the high coherence and excellent shot-to-shot stability both experimentally and numerically. We believe that the presented approach paves the way to outside-lab applications of SC sources in harsh environment.

## Results and Discussion

**Figure 1:**
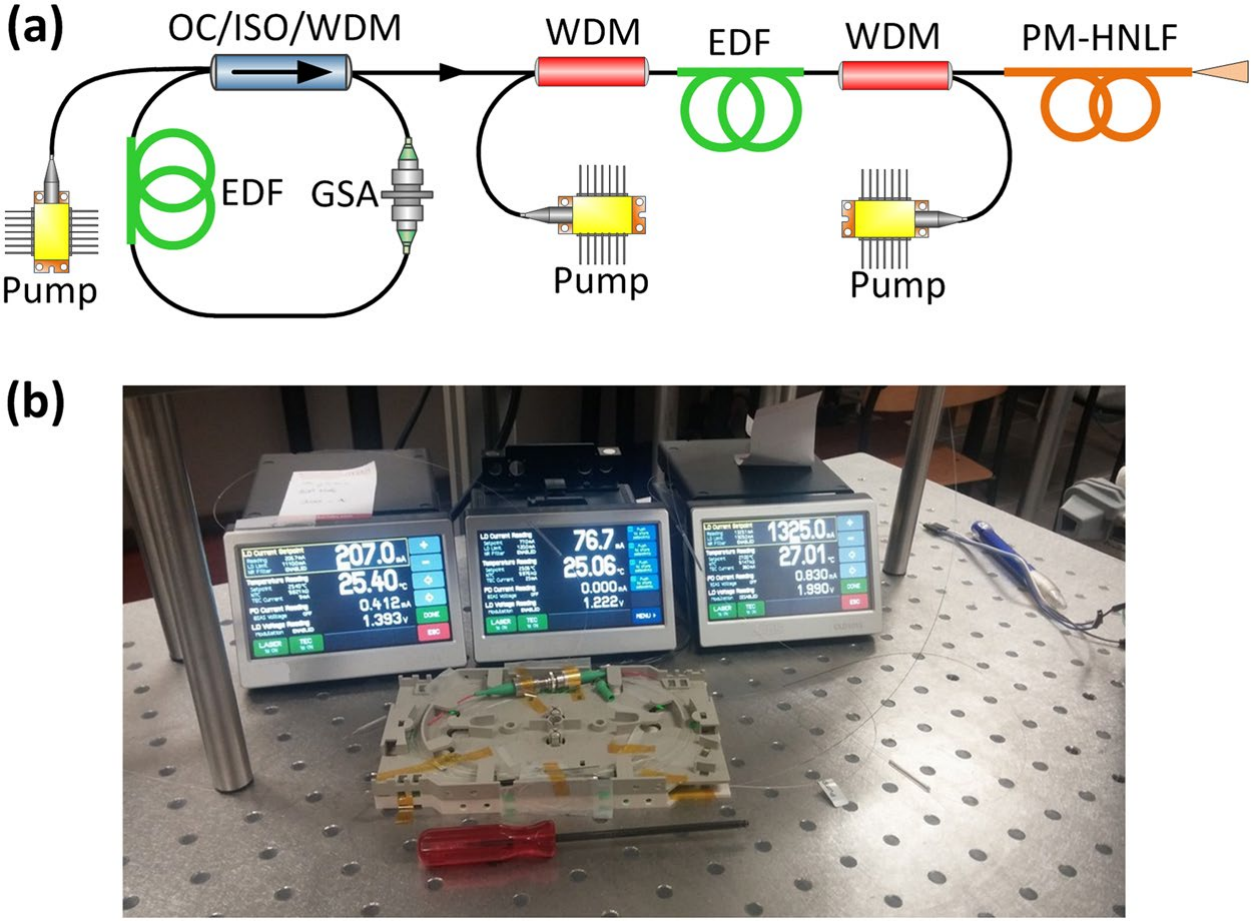
(**a**) Experimental setup of the all-fiber ANDi-SC source. EDF: Erbium-doped fiber. GSA: graphene saturable absorber. OC: output coupler, ISO: isolator, WDM: wavelength division multiplexer, PM-HNLF: polarization maintaining highly nonlinear fiber. (**b**) Photograph of the device running in the lab. The oscillator and amplifier are placed in two standard telecom trays (laser diode drivers are in the back).

**Figure 2:**
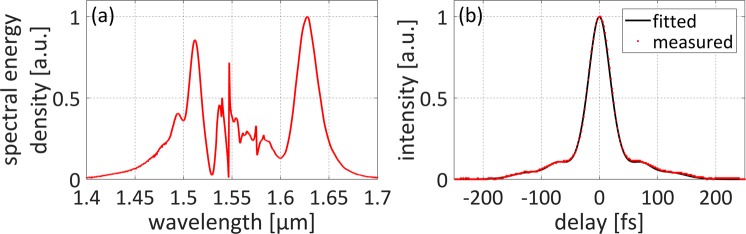
Measured pump pulse characteristics: (**a**) optical spectrum, (**b**) autocorrelation trace (the fitting parameters are gathered in Table 1 in section Methods).

The experimental setup of the coherent supercontinuum source is presented in Fig. 1. The system is built of only three fiber optic components and three pump lasers. An Er-doped fiber laser (EDFL) followed by an amplifier is used as the pump source for SC generation. The EDFL is based on a hybrid component, which comprises an output coupler (OC), isolator (ISO) and wavelength-division multiplexer (WDM) in one integrated package. The oscillator is mode-locked with a graphene saturable absorber (GSA). After amplification, the average output power reaches 116 mW at 45 MHz repetition frequency, which corresponds to a pulse energy of approx. 2.6 nJ. The pulse duration is at the level of 25 fs. The optical spectrum and pulse autocorrelation are depicted in Fig. 2. A small pedestal is visible in the pulse autocorrelation and we performed fitting of the autocorrelation assuming that there are two subpulses that accompany the main pulse as described in Methods. The details on the fiber laser can be found in ref.^22^. The polarization maintaining highly nonlinear fiber (PM-HNLF) used for SC generation is directly spliced to the output of the amplifier. The entire source is based on standard, PM single-mode fibers (PM-SMF) and components. Since the PM-HNLF is a silica fiber with a solid glass core, it is easily spliceable to the PM-SMF with reasonable loss (approx. 2 dB) without using sophisticated techniques incorporating intermediate fibers or expensive filament splicers. It is worth noting that the system works also with only one pumping diode in the amplifier (as described in^22^). The generated supercontinuum is however slightly narrower and does not cover one octave, nevertheless, it also maintains high coherence and all other stability features. The output port of the system can be easily connectorized by splicing a PM pigtail.

**Figure 3:**
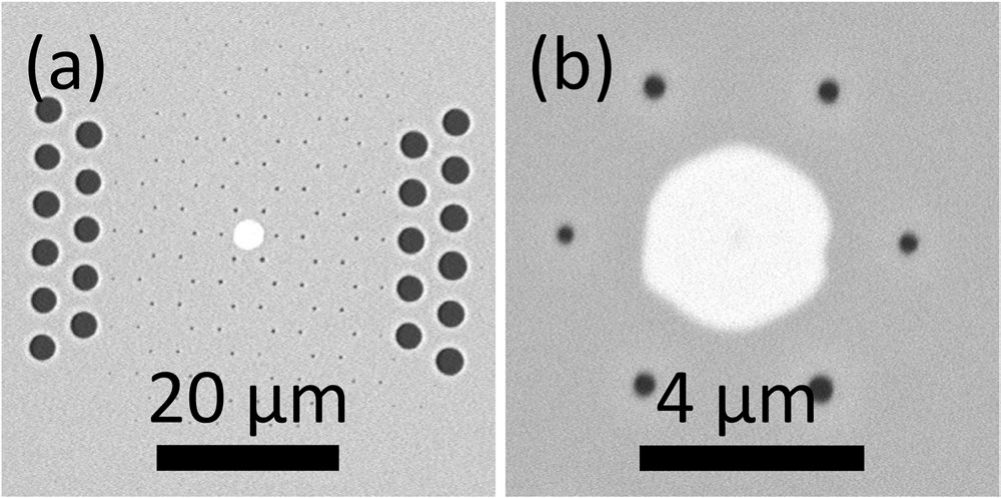
SEM images of microstructured fiber cross-sections: (**a**) core-cladding microstructure, (**b**) zoom on the core area.

**Figure 4:**
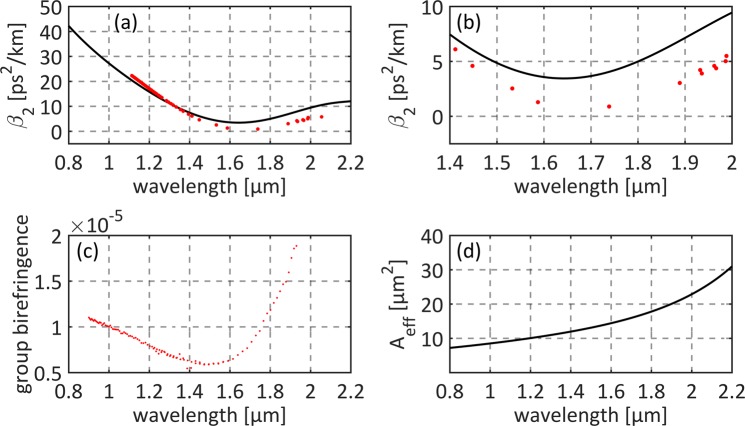
Characteristics of the PM-HNLF: (**a**) group delay dispersion; dotted line: measurement, solid line: calculation based on SEM image, (**b**) group delay dispersion zoom to 1.4–2.0 μm range (**c**) measured group birefringence, (**d**) calculated effective mode area.

The PM-HNLF used for SC generation is a silica microstructured fiber with a germanium doped core, which exhibits all-normal dispersion^12^. Scanning electron microscope (SEM) images of the fiber’s cross section are presented in Fig. 3. The fiber’s preform was stacked with a germanium-doped rod (core) and pure silica rods and capillaries (cladding). The capillaries were grouped symmetrically on two sides of the core (Fig. 3(a)). This alignment allows to induce lattice squeezing during fiber drawing and in consequence to force birefringence. The same approach was successfully applied in^13^. The germanium-doped area (doping level of 18 mol%) has a hexagonal shape. The equivalent ellipse (in terms of normalized second central moments) has the major axis of 3.37 μm and the minor axis of 3.22 μm, showing slight squeeze. The diameter of the first air holes ring is estimated to 6.2 μm and the averaged diameter of the air holes is 0.38 μm. The measured and calculated properties of the fiber are presented in Fig. 4. The comparison of measured and calculated group velocity dispersions (GVD, *β*_2_) is depicted in Fig. 4(a,b). The GVD reaches the minimum value of 0.8 ps^2^/km around 1.7 μm (what corresponds to the maximum chromatic dispersion *D* = −0.5 ps/km/nm). The fiber exhibits group birefringence at the level of 10^−5^, as can be seen in Fig. 4(c). As it will be shown later, this birefringence level allows to maintain linear polarization of the generated SC. The calculated effective mode area keeps below 30 μm^2^ in the entire range of interest, as plotted in Fig. 4(d), and takes the value of 14 μm^2^ at 1.55 μm.

**Figure 5:**
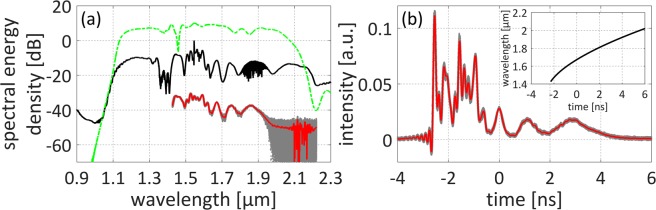
Characteristics of generated SC spectrum: (**a**) comparison of spectrum registered with OSA (black), spectrum calculated with NLSE (green) and spectrum retrieved with DFT: grey dots and red line (single shots and averaged spectrum, respectively); (**b**) temporal traces of 500 shots and their average grey dots and red line (single shots and their average, respectively); the inset shows time-wavelength mapping.

The supercontinuum generation takes place on 73 cm of fiber length. The spectrum covers one octave from 1.1 μm to 2.2 μm as shown in Fig. 5(a) (black line) and the average output power measured directly after the HNLF (without the output pigtail) is 57.2 mW. After splicing an additional connector the power is at the level of 45 mW. The calculated output spectrum is presented in Fig. 5(a) with green line and is in qualitative agreement with measured spectrum in terms of width and flatness. The calculations are based on a self-developed Nonlinear Schrödinger Equation (NLSE) solver. We attribute the low flatness of the generated SC to the non-ideal shape of the input pump pulse. As can be seen Fig. 2(b) and described in Methods, the pulse contains a pedestal which can be fitted as a superposition of three subpulses. It was confirmed in^12^ that the non-ideal shape induces degradation of the flatness. Additionally, the dips around 1.4 μm and 2.2 μm are related to OH absorption bands.

To verify the stability of the source, the SC was characterized with dispersive Fourier transform (DFT) technique, commonly used to investigate the shot-to-shot noise of SC sources^23,24^. Figure 5(b) presents imposed temporal traces of 500 DFT shots (grey dots) and their average (red line). The inset shows time-wavelength mapping applied to restore spectral dependence of individual pulses and their average presented in Fig. 5(a) with grey dots and red line. The wavelength range of DFT is limited by the properties of stretching fiber. The stretching fiber has a zero dispersion wavelength at 1.29 μm, as a result the time-wavelength mapping is not unique below 1.425 μm. On the other hand, the stretching fiber is attenuating beyond 1.9 μm (10 dB at 1.94 μm, >20 dB at wavelengths >2.0 μm) and as a result the long wavelength limit of DFT is around 1.9 μm. The comparison shown in Fig. 5(a) proves that spectrum registered with OSA and obtained with DFT are in excellent agreement in the 1.425–1.9 μm range, which confirms the proper time-wavelength mapping. What can be also seen from Fig. 5 is that the generated SC is characterized by very good stability and very low shot-to-shot fluctuations, comparable to ANDi-SC generated in soft glass fibers^24^. However, in all previous experiments, the fibers were free-space coupled with the pumping sources, which makes them useless in real-life applications.

**Figure 6:**
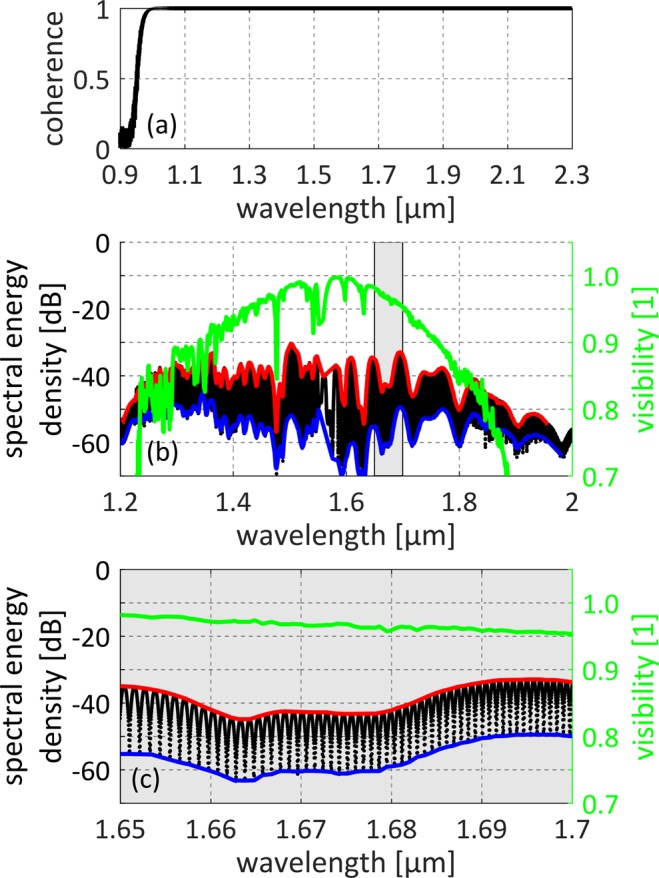
Results of coherence calculations and measurements: (**a**) coherence calculated with NLSE solver, (**b**) measured pulse-to-pulse interference and calculated fringes visibility, (**c**) measured pulse-to-pulse interference and calculated visibility zoomed in 1.65–1.7 μm range.

The coherence properties of the generated SC were investigated both numerically and experimentally. We calculated the spectral coherence g_12_^25^ by performing 500 simulations with random noise (one-photon per mode) using the NLSE solver^25–27^. The result presented in Fig. 6(a) shows that generated spectrum maintains coherence in the entire covered range. To confirm high coherence experimentally, we performed measurements of pulse-to-pulse interference using a fiber-based unequal-path Michelson interferometer^28,29^. The interference pattern of two consecutive pulses of the train is shown in Fig. 6(b,c) with black dots. The shaded area in Fig. 6(b) corresponds to 1.65–1.7 μm range, which is zoomed in Fig. 6(c), to reveal individual fringes. The upper (red) and bottom (blue) envelopes of the spectra were used for calculation of the fringe visibility function (*V*, green), which can be directly referred to coherence^24,28,29^. The measurement confirms that the generated SC is highly coherent. The interference pattern was observed in slightly limited spectral range, due to limited operation range of the 50/50% fiber coupler used in the interferometer setup (originally designed for the 1.55 μm window), nevertheless the interference pattern visibility is higher than 0.7 in 1.23–1.88 μm range. The visibility is also limited by the presence of OH absorption band at 1.89 μm and higher order modes below 1.3 μm. The visibility distribution over the entire observed bandwidth is not flat mainly due to the non-equal splitting ratio of the used fiber coupler for wavelengths >1.7 μm and <1.4 μm.

**Figure 7:**
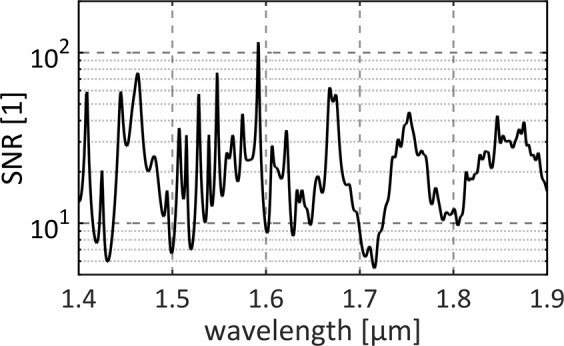
Signal to noise ratio measured with DFT technique in 1.425–1.9 μm range.

**Figure 8:**
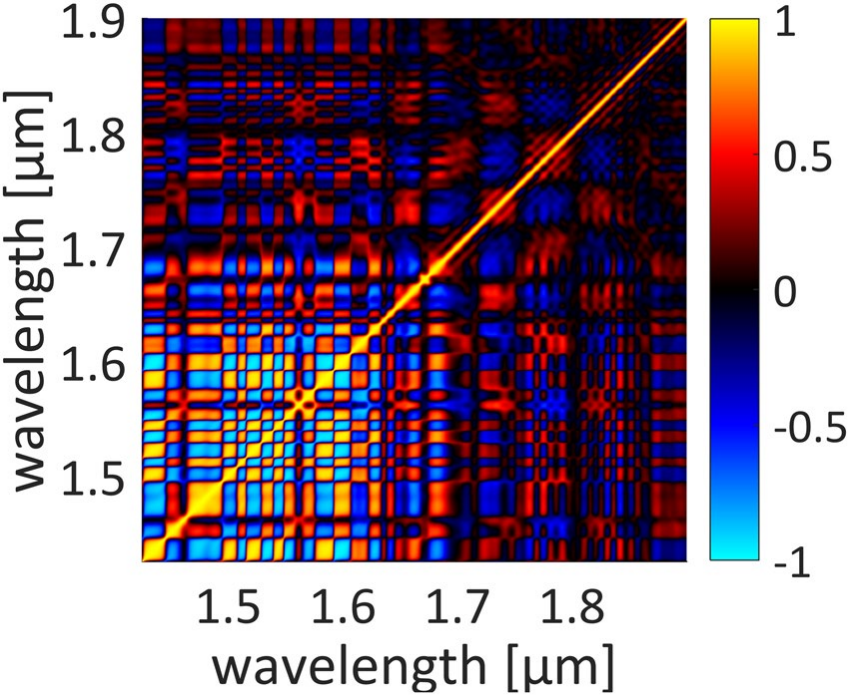
Spectral correlation maps obtained with DFT for the investigated all-fiber ANDi-SC.

We also took advantage of the DFT measurement and calculated the signal to noise ratio (SNR, defined as a ratio between mean and standard deviation at given wavelength) and the correlation maps of the generated SC. Both parameters are often used to analyze shot-to-shot stability of SC sources^24,30^. The SNR vs. wavelength plot is presented in Fig. 7. and the correlation map presented in Fig. 8. The obtained results confirm high signal-to-noise ratio, beyond 10 in almost entire spectral range, with maximum value of ~100. The SNR values are close to obtained for SC generated in soft glass PCF^24^.

The stability, noise and shot-to-shot dynamics of SC generation can be studied by using a wavelength correlation map, which describes how the pairs of wavelengths in the spectrum are correlated with each other. The wavelength correlation function, as defined in^30^, varies between −1 and 1. A positive correlation (red color in Fig. 8) between two chosen wavelengths from the x- and y-axis indicates that the intensities at these wavelengths are bound together (they increase or decrease together). Negative correlation (blue color) means that the pair of wavelengths is anti-correlated: when the intensity of one wavelength increases, the other decreases (and vice versa). The computed correlation map based on experimental data of the generated SC reveals fine structure which is similar to reported earlier^24^. The clearly visible chessboard pattern multiplies the spectral correlation pattern of pump laser line related to wavelength jitter. The correlation map shows that the self-phase modulation (SPM), the stimulated Raman scattering (SRS) and the four-wave mixing (FMW) processes are responsible for spectral broadening. The SRS and FWM processes dominates SPM beyond 1.65 μm as the correlation map is smoothed out for those wavelengths. The mostly checker-pattern-like appearance in the correlation map indicates wavelength jittering present in the supercontinuum, which might result from drifts in the pump laser. Since the entire system is free-running (without any active or passive stabilization of the oscillator or amplifier), we believe that this might be further optimized by applying e.g. thermal stabilization of the laser or improve the 980 nm pump stabilization.

**Figure 9:**
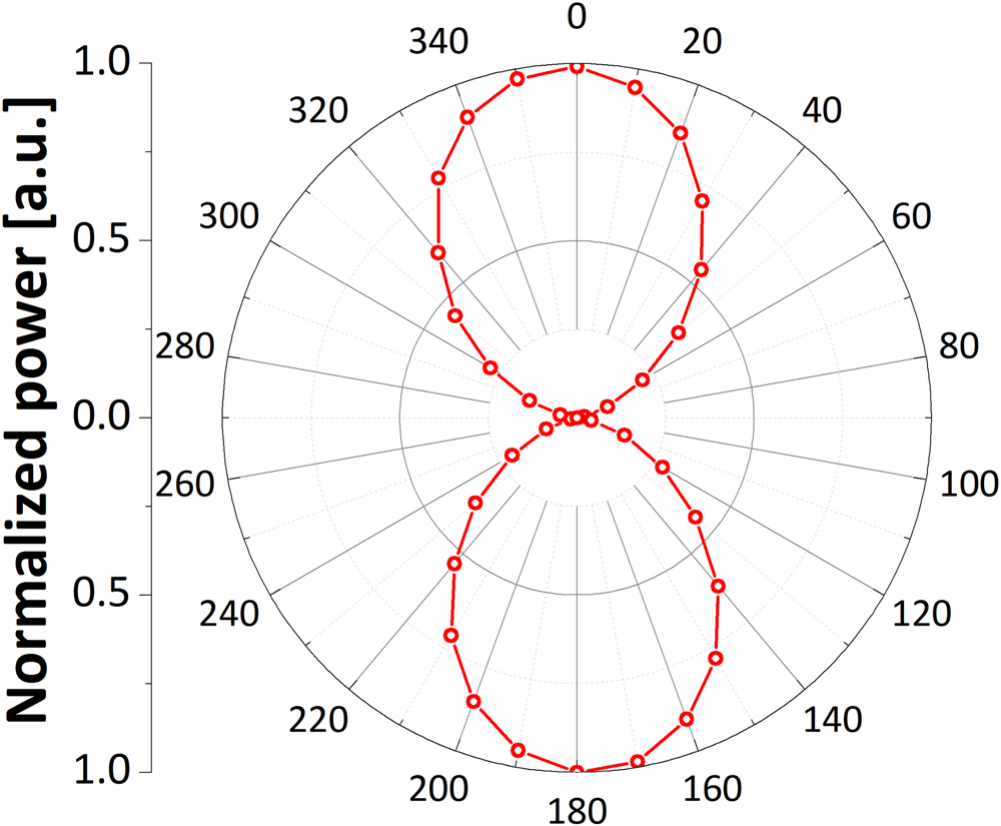
Polar plot indicating the high polarization extinction ratio of the generated SC.

Finally, we characterized experimentally the polarization state of the generated SC spectrum. Figure 9. presents the normalized output power as a function of orientation of a broadband polarizer placed after the SC output. The results confirm that spectrum is linearly polarized with polarization extinction ratio (PER) at the level of 1:57 (~18 dB).

## Methods

### Er-doped fiber laser

All fibers in the laser cavity were spliced using a standard arc-fusion splicer (Fujikura FSM-100P). The EDF is Liekki Er80-4/125-PM and the passive fibers are Fujikura SM15-PS-U25D or equivalent. The output radiation of the laser was characterized with an optical spectrum analyzer (OSA, Yokogawa AQ6375) and an autocorrelator (APE PulseCheck).

### Dispersive fourier transform measurement

The DFT was performed in an unequal-path Michelson interferometer similar to that presented in^24,29^, based on a 50/50 splitter (designed for 1550 nm). As a dispersive medium for DFT, a 500-m-long segment of SM2000 fiber (FIBRAIN Ltd.) was used, which is superior to SMF-28 in terms of long-wavelength transmission. The DFT results were recorded using an OSA (Yokogawa AQ6375) and 6 GHz bandwidth oscilloscope (Agilent Infinuum DSO90604A) coupled with a 13 GHz bandwidth InGaAs photodetector (Discovery Semiconductors DSC2-50S).

### Nonlinear simulations of supercontinuum generation

The calculations were based on solving the NLSE via split-step Fourier method^25–27^ with self-developed software. In the nonlinear simulations we used the fiber properties given in Fig. 4 (group velocity dispersion, and effective mode area). We used calculated group velocity as data are needed for the NLSE solver in a broad range. We also used the fiber loss given in^12^. In order to estimate the pulse shape for nonlinear simulations, we performed fitting of intensity autocorrelation with assumed pulse shape given with Eq (1):

1 $$E(t)={E}^{(A)}\text{sech}(\frac{t}{{T}^{(A)}})+{E}^{(B)}\text{sech}(\frac{t-{t}^{(B)}}{{T}^{(B)}})+{E}^{(C)}\text{sech}(\frac{t-{t}^{(C)}}{{T}^{(C)}})$$

The pulse is described as the sum of three subpulses: main (A) and two additional (B and C). The additional subpulses are introduced to account a small pedestal observed in the autocorrelation trace. The fitting parameters are summarized in Table 1.

**Table 1:** Fitted parameters of the pump pulse.

Subpulse	normalized amplitude E^(i)^ [1]	pulse width T^(i)^ [fs]	pulse delay t^(i)^ [fs]
A	1.0000	16.5	—
B	0.2421	13	72
C	0.1698	27	127

In the simulations, we set the input pulse described with Eq. (1) with parameters given in Table 1. We estimated the average power of input pulse to 72 mW – for this input power, the calculated output power was 57 mW – in agreement with the measurements. This way, we estimated the coupling efficiency to 62% (in agreement with measured splice loss).

## Summary

To summarize, we have reported the first, to our knowledge, entirely fiberized source of coherent and linearly polarized octave-spanning supercontinuum generated in an all-normal dispersion fiber. The silica ANDi fiber is pumped at 1.55 μm by an ultrasimple Er-doped fiber laser, resulting in a broad SC spanning from 1.1 μm to 2.2 μm. The average output power of the SC is at the level of 57 mW. We have confirmed the high coherence of the SC by means of dispersive Fourier transform and spectral interference between consecutive pulses of the SC train. We believe that the excellent coherence properties connected with unprecedented simplicity, compactness, low-cost, and lack of any moving parts makes the presented SC source attractive for practical applications in outside-laboratory conditions.
